# MicroRNA-21 Mediates the Protective Effect of Cardiomyocyte-Derived Conditioned Medium on Ameliorating Myocardial Infarction in Rats

**DOI:** 10.3390/cells8080935

**Published:** 2019-08-19

**Authors:** Chih-Hung Chen, Shu-Yuan Hsu, Chien-Chih Chiu, Steve Leu

**Affiliations:** 1 Divisions of General Medicine, Department of Internal Medicine, Kaohsiung Chang Gung Memorial Hospital and Chang Gung University College of Medicine, Kaohsiung 833, Taiwan; 2 Graduate Institute of Adult Education, National Kaohsiung Normal University, Kaohsiung 802, Taiwan; 3 Institute for Translational Research in Biomedicine, Kaohsiung Chang Gung Memorial Hospital, Kaohsiung 833, Taiwan; 4 Department of Anatomy, Graduate Institute of Biomedical Sciences, College of Medicine, Chang Gung University, Taoyuan 333, Taiwan; 5 Department of Biotechnology, College of Life Science, Kaohsiung Medical University, Kaohsiung 807, Taiwan

**Keywords:** conditioned medium, oxygen-glucose-deprivation, microRNA-21, cardiomyocyte

## Abstract

Conditioned medium derived from ischemic myocardium improves rodent cardiac function after myocardial infarction. Exosomal miRNA-mediated intercellular communication is considered to mediate the protective effect of conditioned medium against ischemic injury. Oxygen–glucose-deprivation (OGD)-treated cardiac cells and a rat model with acute myocardial infarction (AMI) were applied. The expression profiles of myocardial-disease-associated miRNAs in cardiomyocytes, cardiac fibroblasts, ventricular myocardium, and conditioned medium derived from cardiomyocytes under ischemic stresses were analyzed. Primary cultured cell model and a rat model with myocardial infarction were applied to examine the role of miRNA in regulating cardiomyocyte apoptosis, fibroblast activation, immune cell infiltration, and myocardial infarction. Results showed that expression levels of miR-21 in cardiomyocytes, cardiac fibroblasts, and conditioned medium (CM) derived from cardiomyocytes were up-regulated with OGD treatment. With the depletion of miR-21, the protective effect of CM on cardiomyocytes against oxidative stress, enhanced fibroblast activation, and promotion of angiogenesis in endothelial cells were reduced. Administration of CM reduced the infarcted size and immune cell infiltration in myocardium of rats with AMI, while depletion of miR-21 reduced the effect of CM. In conclusion, miR-21 plays a role in intercellular communication among ischemic cardiac cells. The expression of miR-21 is important for the protective effect of conditioned medium against myocardial infarction.

## 1. Introduction

Acute myocardial infarction (AMI) is caused by coronary artery occlusion and characterized by a loss of viable myocardium. After myocardial infarction, interstitial fibrosis in cardiac remodeling further deteriorates pump function and induces arrhythmias and sudden death [1,2]. Clinical findings have shown that AMI patients with prior repeated attacks of angina have a better prognosis and survival rate than those without repeated angina [3]. Subsequent studies in large experimental animal models also confirmed the protective function of repeated brief ischemia/reperfusion before AMI, which is termed “ischemic preconditioning” (IPC) [4,5]. In recent years, the cross-talk between different types of cardiac cells has been shown to play important roles in cardiac remodeling [6,7,8,9]. In addition, the benefits of IPC could be caused by the interaction between cells, including direct cell–cell contact and the paracrine effects. [10,11]. Our recent study showed that conditioned medium collected from ischemic myocardium had a protective function against myocardial infarction in rats [12], however, the major components which mediate the protective intercellular communication among cardiac cells remain unknown. 

MicroRNAs (miRNAs) are short, regulatory RNAs that act as negative regulators of gene expression by inhibiting mRNA translation or by promoting mRNA degradation [13,14]. Growing evidence shows that miRNAs are extensively involved in the pathogenesis of heart diseases, including cardiac hypertrophy, dilated cardiomyopathy, and arrhythmia [15,16,17,18,19]. Although the miRNA expression profiles in both animals and humans with heart diseases have been revealed in several studies [17,20,21,22], the in situ expression patterns of miRNAs in failed hearts have not been fully determined. Whether these miRNAs are synthesized in cardiomyocytes, cardiac fibroblasts, or endothelial cells during the progression of cardiac remodeling is still not fully clear, and needs to be clarified. Recently, exosomal miRNAs have been demonstrated as another functional signaling messenger between adjacent cells [23,24,25]. Previous studies have found that miR143/145 released from shear-stress-treated endothelial cells could be transferred into adjacent smooth muscle cells (SMC) and regulate the proliferation and contraction of SMC [26]. Meanwhile, exosomal transfer of miR-320 from cardiomyocytes of type 2 diabetic rats also regulates angiogenic activity of surrounding endothelial cells [25]. It is reasonable to suggest that exosomal miRNA may play roles to mediate the protective effects of conditioned medium on ameliorating myocardial injury after acute myocardial infarction. 

In the present study, we analyzed the expression profile of myocardial-disease-associated miRNAs in cardiomyocytes, cardiac fibroblasts, ventricular myocardium, and conditioned medium derived from cardiomyocytes, and then examined the effect of ischemic stress on the expression levels of the miRNAs. An in vitro cultured cell model and an in vivo animal model with myocardial infarction were applied to further determine the role of miRNAs in regulating cardiomyocyte apoptosis, fibroblast activation, immune cell infiltration, and myocardial infarction. 

## 2. Materials and Methods

### 2.1. Ethics

All animal experimental procedures were approved by the Institute of Animal Care and Use Committee at Kaohsiung Chang Gung Memorial Hospital (No. 2013062702) and performed in accordance with the Guide for the Care and Use of Laboratory Animals (NIH publication No. 85-23, National Academy Press, Washington, DC, USA, revised 1996).

### 2.2. Animals and Induction of Acute Myocardial Infarction in Rats

All experimental animals in this study were housed in an Association for Assessment and Accreditation of Laboratory Animal Care International (AAALAC)-certified animal facility in our hospital with controlled temperature and light cycles (24 °C and 12/12 light cycle). Experimental procedures were performed in pathogen-free, adult male Sprague–Dawley (SD) rats, weighing 275–300 g (Charles River Technology, BioLASCO Taiwan Co., Ltd., Taiwan). SD rats were anesthetized by intraperitoneal injections of chloral hydrate (35 mg/kg). Rats were placed in a supine position on a warming pad at 37 °C after being shaved on the chest and then intubated with positive-pressure ventilation (180 mL/min) with room air using a small animal ventilator (SAR-830/A, CWE, Inc., Ardmore, PA, USA). Under sterile conditions, the heart was exposed via a left thoracotomy at the level of the fifth intercostal space. AMI induction was performed via left coronary artery ligation (LCAL) 2 mm below the left atrium with a 7-0 Prolene suture. Regional myocardial ischemia was observed by a rapid discoloration over the anterior surface of the LV, together with the development of akinesia and dilatation over the at-risk area. After induction of AMI, 75 μL of conditioned medium or normal medium was injected into the anterior wall of left ventricle. 

### 2.3. Culturing of Cardiac Cells

**Figure 1:**
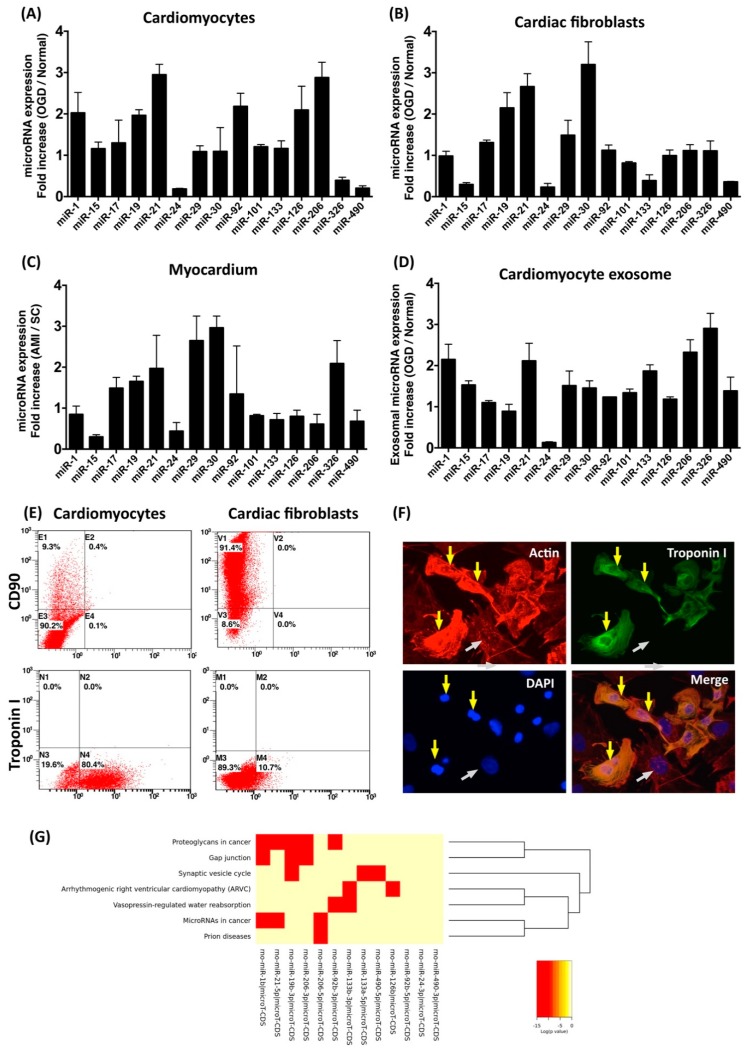
Expression profile of myocardial-disease-associated microRNA in myocardium, cardiac fibroblasts, cardiomyocytes, and cardiomyocyte-derived conditioned medium. (**A**) Relative expression level of miRNAs in cardiomyocytes incubated under oxygen–glucose deprivation (OGD) and normal (normoxia with serum and glucose supply) conditions for 6 h. (**B**) Relative expression level of miRNAs in cardiac fibroblasts incubated in OGD and in normal condition for 6 h. (**C**) Relative expression level of miRNAs in ventricular myocardium with and without induction of acute myocardium infarction. (**D**) Relative expression level of miRNAs in conditioned medium derived from cardiomyocytes with and without OGD treatment. (**E**) Flow cytometric assessment with antibodies against CD90 (cardiac fibroblast marker) and troponin I (cardiomyocyte marker) to examine the population of isolated cardiac cells. (**F**) Immunofluorescent staining with antibodies against troponin I to identify isolated cardiomyocytes. Yellow arrows indicate cardiomyocytes with troponin I expression. White arrows indicate non-cardiomyocytes. (**G**) Heatmap generated from DIANA-miRPath 3.0 to show KEGG pathways targeted by OGD-regulated miRNAs in cardiomyocytes. Six hours after OGD treatment or acute myocardial infarction (AMI) induction, total RNAs were extracted from cells, myocardium, or conditioned medium, followed by real-time RT-PCR with specific microRNA primers. n = 3 for each group.

To prepare neonatal cardiomyocytes and cardiac fibroblasts from 2 day old Sprague–Dawley rats, ventricles were isolated and then digested with collagenase (0.4 mg/mL) and pancreatin (0.6 mg/mL) in 116 mM NaCl, 20 mM HEPES (pH 7.35), 0.8 mM NaH_2_PO_4_, 5.6 mM glucose, 5.4 mM KCl, and 0.8 mM MgSO_4_. Cells were recovered by centrifugation and then resuspended in plating medium (80% DMEM, 20% M199, 15% fetal bovine serum (FBS), 100 U/mL of penicillin and streptomycin) and pre-plated on gelatin pre-coated 60 mm culture dishes. After pre-plating for 3 h, adherent cardiac fibroblasts were cultured in DMEM + 10% FBS, while non-adherent cardiomyocytes were transferred to new plates for further culturing. For cardiomyocyte selection, arabinoside (10 nM) was added into the medium to inhibit the potential proliferation of fibroblasts. Twenty-four hours after plating, cells were washed with the medium and used for experiments. The purity of cells was confirmed by flow cytometry with antibodies against Troponin I and CD90 (Figure 1E). For hypoxia (3% O_2_), cardiac fibroblasts were incubated in a hypoxia chamber (HeraCell 150i, Thermo Scientific, Waltham. MA, USA), while normoxic (21% O_2_) conditions were used for culturing cells in the normal condition. Inhibitor of miR-21-5p: 5- UAGCUUAUCAGACUGAUGUUGA-3 (10, 20, 40 nM, AM10206, Ambion, Austin, TX, USA) and negative control oligo (20 nM, Anti-miR miRNA inhibitor negative control, AM17010, Ambion, Austin, TX, USA) were transfected into cardiomyocytes with X-tremeGENE siRNA transfection reagent (Roche, Basel, Switzerland).

### 2.4. Collection of Exosomes

To examine the level of exosomal miR-21 in conditioned medium derived from cardiomyocytes with ani-miR-21 inhibitor transfection, exosomes were purified from conditioned medium via a combination of ultrafiltration and ultracentrifugation. Initially, culture medium was collected from cardiac cells and then centrifuged at 15,000× *g* for 20 min to remove debris. The resulting cell-free medium was concentrated by ultrafiltration using an Amicon stirred cell Model 8200 with a molecular weight cutoff membrane of 500,000 Daltons (Millipore, Billerica, MA, USA). This concentrated material was then ultra-centrifuged at 100,000× *g* for 90 min at 4 °C to generate an exosome pellet. The pellet was resuspended and washed twice with PBS.

### 2.5. Quantitative Reverse Transcription Polymerase Chain Reaction

Quantitative RT-PCR reactions were completed on a 7500 Fast Real Time PCR system (Life Technologies, Carlsbad, CA, USA). The relative quantity of the target miRNA was normalized to an endogenous gene (U6 snRNA). Total RNA from cells, myocardium, conditioned medium, and exosomes were isolated using a mirVana total RNA isolation kit (Life Technologies) according to the manufacturer’s guidelines. For miRNA expression validation, total RNA (10 ng) was converted into cDNA using specific miRNA primers and a miRNA reverse transcription kit (Life Technologies). The selection of miRNAs was according to previous studies in which the involvement of miRNAs in myocardial infarction was reported [27,28,29]. The expression level of miRNAs was calculated with the 2^−ΔΔCt^ method. Samples were run in triplicate and three independent experiments were performed. Data are presented as mean ± SD. The Kyoto Encyclopedia of Genes and Genome (KEGG) pathways linking to miRNAs was analyzed with the DIANA-miRPath V3.0 web server [30]. 

### 2.6. Histopathological and Immunostaining

For immunofluorescent staining, isolated myocardium was mounted in OCT and used for preparing cryosections, while cardiomyocytes were seeded on coverslips for 24 h. Sections or coverslips were fixed and permeated with ice-cold acetone or 4% paraformaldehyde with 0.5% Triton X-100, and then incubated with antibodies against CD90, Troponin I, CD68, or CD11b at 4 °C overnight. Coverslips or slides were then incubated with Alex488 or Alex594-conjugated goat anti-mouse or rabbit IgG (Invitrogen, Carlsbad, CA, USA). After counterstaining with DAPI, samples were examined under a fluorescent microscope. To analyze the extent of collagen synthesis and deposition, cardiac paraffin sections (6 µm) at 3 mm intervals were stained with Masson’s Trichrome. Masson’s Trichrome staining was performed according to the protocol provided by the manufacturer. In brief, sections were firstly fixed in Bouin’s solution. After incubation in Weigert’s Iron Hematoxylin solution, the slides were stained with Biebrich Scarlet-Acid Fuchsin and Aniline Blue, and then dehydrated in ethanol and xylene. 

### 2.7. Western Blot

Equal amounts (10–30 μg) of protein extracts from cells or myocardium were loaded and separated by SDS-PAGE using 8–10% acrylamide gradients. Following electrophoresis, the separated proteins were transferred electrophoretically to a polyvinylidene difluoride (PVDF) membrane (Amersham Biosciences, Piscataway, NJ, USA). Nonspecific proteins were blocked by incubating the membrane in blocking buffer (5% nonfat dry milk in T-TBS containing 0.05% Tween 20) overnight. The membranes were incubated with antibodies against cleaved caspase-3 (c-Csp 3) (1:1000, Cell Signaling, Danvers, MA, USA), cleaved poly (ADP-ribose) polymerase (c-PARP) (1:1000, Cell Signaling), TGF-β (1:500 Cell Signaling), phosphorylated Smad2 (1:1000 Cell Signaling), Bax (1:1000, Abcam, Cambridge, MA, USA), and β-actin (1:10,000, Millipore) for 1 h at room temperature. Signals were detected with HRP-conjugated goat anti-mouse or goat ant-rabbit with ECL (Perkin Elmer, MA, USA).

### 2.8. Statistical Analysis 

Data have been expressed as mean values (mean ± SD). The significance of differences among the groups was evaluated using one-way ANOVA, followed by Bonferroni multiple comparison post hoc test. Statistical analysis was performed using Prism 6 statistical software (GraphPad Software, La Jolla, CA, USA). A probability value < 0.05 was considered statistically significant.

## 3. Results

### 3.1. Oxygen–Glucose Deprivation Regulates microRNA Expression Profiles in Cardiac Cells

Recent studies have demonstrated that induction or repression of certain miRNAs after myocardial infarction triggers downstream events in cardiac fibroblasts and cardiomyocytes, while interference with endogenous miRNAs expression regulates cardiac function [27]. To determine the effect of ischemic stress on regulating miRNA expression profiles in different types of cardiac cells, cardiomyocytes and cardiac fibroblasts were isolated form neonatal rat ventricular myocardium and cultured in specific culturing medium for further purification. To simulate ischemic stress in vitro, isolated cardiomyocytes and cardiac fibroblasts were incubated in oxygen–glucose deprivation (OGD) condition for 6 h. After OGD treatment, total RNA was extracted, followed by real-time RT-PCR to determine the relative expression levels of the selected miRNAs. The selection of miRNAs was according to previous studies in which the involvement of miRNAs in myocardial infarction was reported [27,28,29]. Compared to those in normal cardiomyocytes, expression levels of miR-1, miR-19, miR-21, miR-92, miR-126, and miR-206 were higher in cardiomyocytes with OGD treatment (Figure 1A). In cardiac fibroblasts, the expression levels of miR-19, miR-21, and miR-30 were upregulated by OGD (Figure 1B). In addition to isolated cardiomyocytes and cardiac fibroblasts, the miRNA expression profiles in ischemic ventricular myocardium were also examined. Compared to those in normal myocardium, the expression levels of miR-17, miR-19, miR-21, miR-29, miR-30, and miR-126 were up-regulated in ischemic myocardium (Figure 1C). In addition to intracellular miRNAs, the expression levels of exosomal miRNA within conditioned medium derived from OGD-treated cardiomyocytes were also examined. Results indicated that the expression levels of miR-1, miR-21, miR-133, miR-206, and miR-326 in conditioned medium were upregulated by OGD (Figure 1D). Results from bioinformatic analysis also showed that several pathways associated with cardiac function and myocardial disease, such as gap junction and arrhythmogenic right ventricular cardiomyopathy, were potentially targeted by OGD-regulated miRNAs in cardiomyocytes (Figure 1G). Among miRNAs of which the expression levels were regulated by OGD, the elevation of miR-21 expression was observed in OGD cardiomyocytes, OGD cardiac fibroblasts, ischemic myocardium, and in conditioned medium derived from OGD-treated cardiomyocytes. 

### 3.2. Inhibition of miR-21 Reduced the Protective Effect of Cardiomyocyte-Derived Conditioned Medium Cardiomyocytes Against Oxidative Stress

**Figure 2:**
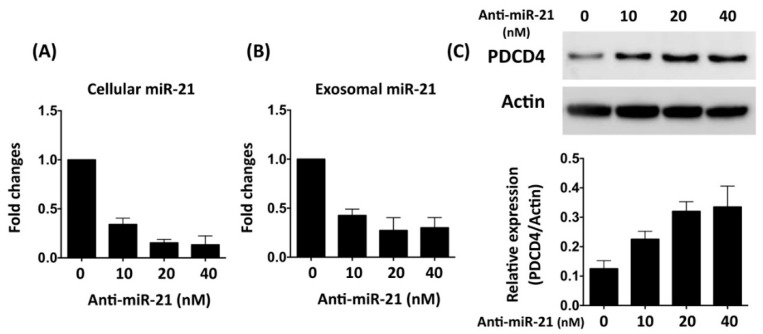
Reduced expression of exosomal miR-21 through transfection of anti-miR-21 in rat primary cardiomyocytes. (**A**) Real-time RT-PCR examination of expression levels of cellular miR-21 in neonatal cardiomyocytes 24 h after delivery of anti-miR-21 (**B**). Real-time RT-PCR examination of expression levels of exosomal miR-21 in neonatal cardiomyocytes 24 h after delivery of anti-miR-21. (**C**) Expression levels of PDCD4, the target of miRNA, in cardiomyocytes transfected with anti-miR-21. n = 3 for each group.

**Figure 3:**
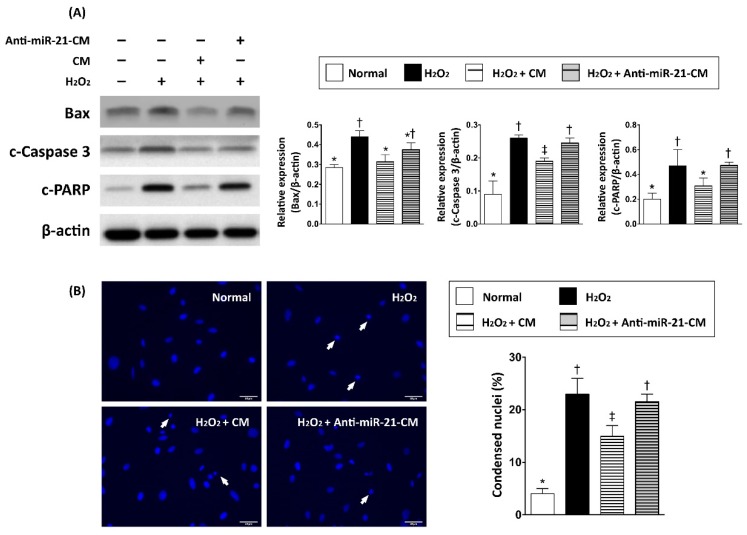
Inhibition of miR-21 reduced the protective effect of cardiomyocyte-derived conditioned medium on cardiomyocytes against oxidative stress. (**A**) Western blots to determine the expression levels of apoptosis-associated proteins, including Bax, cleaved caspase 3 (c-Caspase 3), and cleaved Poly (ADP-ribose) polymerase (c-PAPR), in neonatal cardiomyocytes treated with hydrogen peroxide (50 μM of H_2_O_2_) and cardiomyocyte-derived conditioned medium. (**B**) Nuclear staining with DAPI to observe the condensed nuclei in apoptotic cells. CM, conditioned medium derived from OGD-treated cardiomyocyte. Anti-miR-21-CM, conditioned medium derived from cardiomyocyte pre-transfected with anti-miR-21 (20 nM). Groups with different symbols (*, †, ‡), *p* < 0.05. n = 6 for each group.

To further examine the role of miR-21 in conditioned medium, anti-miR miRNA inhibitor against miR-21-5p was transfected into neonatal cardiomyocyte to inhibit the function of miR-21. Real-time RT-PCR was applied to examine the expression level of miR-21 in cells and secreted exosomes (Figure 2A,B). Previous studies have indicated that miR-21 protects cardiomyocytes against apoptosis through targeting Programmed Cell Death 4 (PDCD4) [31,32]. To evaluate the efficacy of miR-21 inhibitor in inhibiting miR-21 function, the protein expression level of PDCD4 was examined. Results showed that not only was the expression of PDCD4 increased with miR-21 inhibitor transfection (Figure 2C), expression levels of miR-21 in both cardiomyocytes and conditioned medium were also decreased (Figure 2A,B), indicating the miR-21 inhibitor had effects on regulation of the expression level and function of miR-21. To further determine the role of miR-21 in conditioned medium (CM), oxidative-stress-induced cellular apoptosis in primary cardiomyocytes with hydrogen peroxide treatment was utilized to evaluate the protective effect of CM derived from OGD-treated cardiomyocytes. Primary neonatal cardiomyocytes were cultured in cultured medium with CM and normal medium (1:1) for 24 h, and then incubated with hydrogen peroxide (H_2_O_2_, 50 μM) for 24 h. Results from western blots showed that the expression levels of apoptotic proteins, including Bax, cleaved caspase-3, and cleaved PARP, were upregulated by hydrogen peroxide treatment (Figure 3A), indicating the induction of apoptosis by oxidative stress. Fluorescent microscope observation of DAPI-stained nuclei also indicated that the number of condensed nuclei, an apoptotic index, was increased by the oxidative insult (Figure 3B). In cells treated with CM, the expression levels of apoptotic proteins and number of condensed nuclei were reduced, while CM collected from cardiomyocytes pre-transfected with miR-21 inhibitor showed decreased function in protecting cardiomyocytes against oxidative-stress-induced apoptosis (Figure 3A,B).

### 3.3. Inhibition of miR-21 Reduced the Effect of Conditioned Medium on Activation of Cardiac Fibroblasts

**Figure 4:**
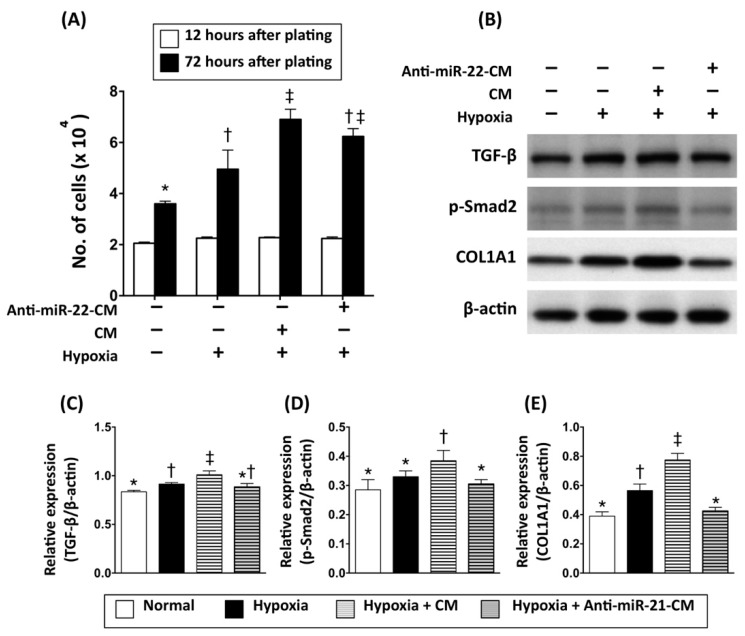
Inhibition of miR-21 reduced the effect of conditioned medium on activation of cardiac fibroblasts. (**A**) Cell number counting to determine the proliferative rate of cardiac fibroblasts incubated with hypoxia (3% O_2_) and in conditioned medium derived from OGD-treated cardiomyocytes. (**B**) Western blots to examine the expression levels of fibrosis signaling proteins (TGF-β, phosphorylated Smad2) and collagen deposition (collagen typ1 1 alpha 1, COL1A1) in primary cardiac fibroblasts incubated with hypoxia and in conditioned medium derived from OGD-treated cardiomyocytes. (**C**) Quantitative data represent the ratio of TGF-β expression to β-actin expression. (**D**) Quantitative data represent the ratio of phosphorylated Smad2 to β-actin expression. (**E**) Quantitative data represent the ratio of COL1A1 to β-actin expression. CM, conditioned medium derived from OGD-treated cardiomyocytes. Anti-miR-21-CM, conditioned medium derived from cardiomyocytes pre-transfected with Anti-miR-21 (20 nM). Groups with different symbols (*, †, ‡), *p* < 0.05. n = 6 for each group.

MiR-21 has been demonstrated to be involved in cardiac fibrosis in failing hearts. To further examine the role of miR-21 in CM on regulating fibroblasts proliferation and fibrotic gene expression, CM derived from OGD-treated cardiomyocytes with or without miR-21 inhibitor pre-transfection was applied to neonatal cardiac fibroblast with hypoxia. Results showed that the proliferative rate of cardiac fibroblasts was increased by hypoxia (3% of O_2_) and further enhanced by CM. However, the reduction of miR-21 through pre-transfection of anti-miR-21 inhibitor did not significantly decrease the CM-induced cellular proliferation in cardiac fibroblast (Figure 4A). Examinations of fibrotic gene expression also showed that expression levels of TGF-β, phosphorylated-Smad2, and collagen type 1 alpha 1 (COL1A1) were increased by hypoxia and cardiomyocyte-derived CM. Of interest, unlike the effect on proliferation of fibroblasts, the pre-transfection of miR-21 inhibitor abolished the effects of CM on upregulating expression of fibrosis-associated genes (Figure 4B).

### 3.4. Inhibition of miR-21 Reduced the Effect of Conditioned Medium on Angiogenic Enhancement in Endothelial Cells

**Figure 5:**
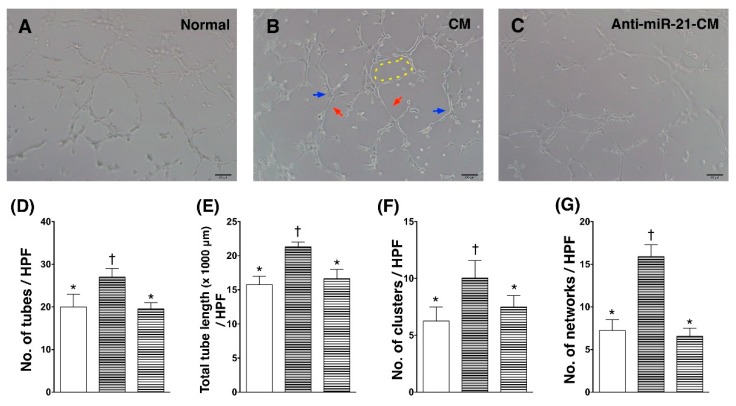
Inhibition of miR-21 reduced the effect of conditioned medium on angiogenic enhancement in endothelial cells. (**A**) Matri-gel analysis on normal control human umbilical vein endothelial cells (HUVEC). (**B**) Matri-gel analysis on HUVECs incubated with conditioned medium derived from OGD-treated cardiomyocytes. (**C**) Matri-gel analysis on HUVECs incubated with conditioned medium derived from OGD-treated cardiomyocytes with anti-miR-21 pre-transfection. (**D**) Calculation of tube formation (red arrows). (**E**) Calculation of total tube length. (**F**) Calculation of cluster formation (blue arrows). (**G**) Calculation of network formation (yellow dotted line). CM, conditioned medium derived from OGD-treated cardiomyocytes. Anti-miR-21-CM, conditioned medium derived from cardiomyocytes pre-transfected with anti-miR-21 (20 nM). Groups with different symbols (*, †), *p* < 0.05. n = 6 for each group.

The neovascularization after ischemic injury plays critical roles in the functional and structural preservation of myocardium after myocardial infarction. To examine the role of miR-21 in CM on modulating angiogenic activity in endothelial cells, human umbilical vein endothelial cells (HUVEC) were incubated in CM derived from OGD-treated cardiomyocytes for 24 h and applied to matri-gel analysis. Results showed that the in vitro angiogenic activity assessments of HUVECs, including number of tubes, number of clusters, number of networks, and total tube length, were increased by CM, while depletion of miR-21 in CM reduced the effect of CM on enhancing angiogenesis in HUVECs (Figure 5). 

### 3.5. Inhibition of miR-21 Reduced the Effect of Conditioned Medium on Ameliorating Myocardial Injury in Rats with Acute Myocardial Infarction

**Figure 6:**
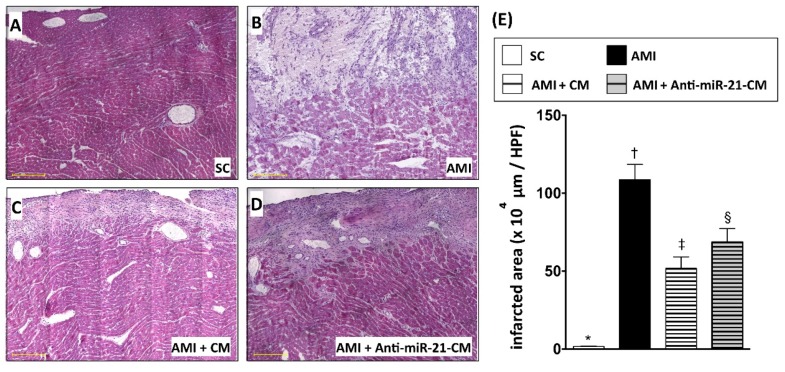
Inhibition of miR-21 reduced the effect of conditioned medium on ameliorating myocardial infarction. (**A**) Histopathological examination on sham-control myocardial section with hematoxylin and eosin (H&E) staining. (**B**) H&E staining for myocardial section from rats with AMI induction. (**C**) H&E staining for myocardial section from rats with AMI induction and injection of conditioned medium OGD-treated cardiomyocytes. (**D**) H&E staining for myocardial section from rats with AMI induction and injection of conditioned medium derived from OGD-treated cardiomyocytes with anti-miR-21 pre-transfection. (**E**) Calculation of infarcted area in myocardium. SC, sham control. AMI, acute myocardial infarction. CM, conditioned medium derived from OGD-treated cardiomyocytes. Anti-miR-21-CM, conditioned medium derived from cardiomyocytes pre-transfected with Anti-miR-21 (20 nM). Groups with different symbols (*, †, ‡, §), *p* < 0.05. n = 8 for each group. Scale bar in left lower corner indicated 200 μm.

**Figure 7:**
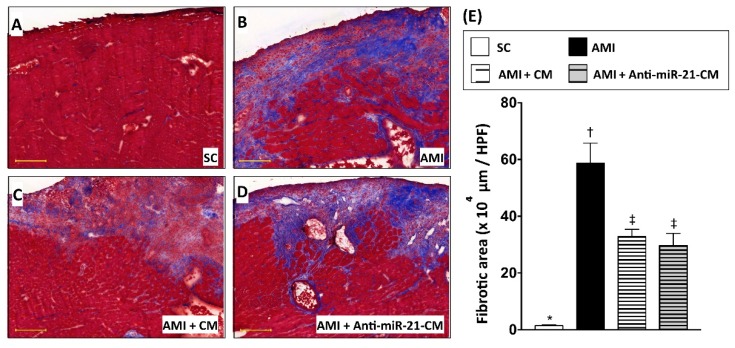
Conditioned medium derived from cardiomyocytes with oxygen–glucose deprivation decreased myocardial collagen deposition in rats with myocardial infarction. (**A**) Histopathological examination on sham control myocardial section with Masson’s trichrome (MTC) staining. (**B**) MTC staining for myocardial section from rats with AMI induction. (**C**) MTC staining for myocardial section from rats with AMI induction and injection of conditioned medium derived from OGD-treated cardiomyocytes. (**D**) MTC staining for myocardial section from rats with AMI induction and injection of conditioned medium derived from OGD-treated cardiomyocytes with anti-miR-21 pre-transfection. (**E**) Calculation of collagen disposition area in myocardium. SC, sham control. AMI, acute myocardial infarction. CM, conditioned medium derived from OGD-treated cardiomyocytes. Anti-miR-21-CM, conditioned medium derived from cardiomyocytes pre-transfected with anti-miR-21. Groups with different symbols (*, †, ‡), *p* < 0.05. n = 8 for each group. Scale bar in left lower corner indicates 200 μm.

To directly evaluate the effect of CM in protecting myocardium against ischemic injury, CM derived from OGD-treated cardiomyocytes was applied to a rat model with acute myocardial infarction (AMI). After ligation of left anterior descending artery (LAD), CM was immediately injected into the ischemic region of myocardium. Twenty-eight days after induction of AMI and CM administration, ventricular myocardium was isolated for histological and molecular biochemical examination. Results from both hematoxylin and eosin (H&E) staining (Figure 6) and Masson’s trichrome (MTC) staining (Figure 7) indicated that the myocardial infarcted area and collagen deposition were reduced by administration of CM. Furthermore, CM collected form miR-21 inhibitor pre-transfected cardiomyocytes showed less efficacy in reducing infarct size. However, the effect of CM on reducing collagen deposition was not affected by miR-21 inhibitor pre-transfection. 

### 3.6. Inhibition of miR-21 Reduced the Effect of Conditioned Medium on Reducing Immune Cell Infiltration in Myocardium with Acute Myocardial Infarction in Rats

**Figure 8:**
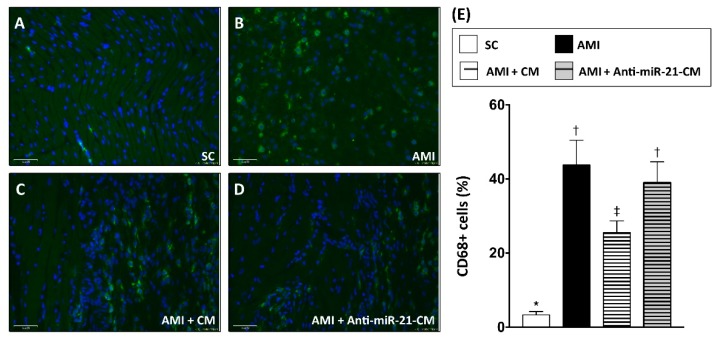
Inhibition of miR-21 reduced the effect of conditioned medium on ameliorating myocardial infiltration of CD68+ macrophages. (**A**) Immunofluorescent staining to detect the distribution of CD68+ macrophages in sham control myocardial section. (**B**) Immunofluorescent staining to detect the distribution of CD68+ macrophages in myocardial section from rats with AMI induction. (**C**) Immunofluorescent staining to detect the distribution of CD68+ macrophages in myocardial section from rats with AMI induction and injection of conditioned medium derived from OGD-treated cardiomyocytes. (**D**) Immunofluorescent staining to detect the distribution of CD68+ macrophages in myocardial section from rats with AMI induction and injection of conditioned medium derived from OGD-treated cardiomyocytes with anti-miR-21 pre-transfection. (**E**) Calculation of CD68+ cells in myocardium. SC, sham control. AMI, acute myocardial infarction. CM, conditioned medium derived from OGD-treated cardiomyocyte. Anti-miR-21-CM, conditioned medium derived from cardiomyocyte pre-transfected with anti-miR-21 (20 nM). Groups with different symbols (*, †, ‡), *p* < 0.05. n = 8 for each group. Scale bar in left lower corner indicates 50 μm.

**Figure 9:**
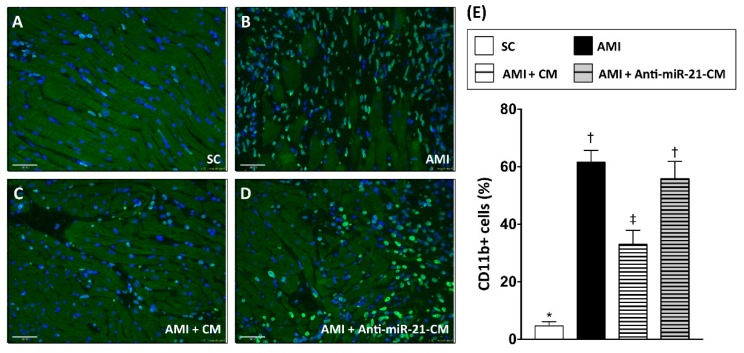
Inhibition of miR-21 reduced the ameliorating effect of conditioned medium on myocardial infiltration of CD11b+ immune cells. (**A**) Immunofluorescent staining to detect the distribution of CD11b+ immune cells in sham control myocardial section. (**B**) Immunofluorescent staining to detect the distribution of CD11b+ cells in myocardial section from rats with AMI induction. (**C**) Immunofluorescent staining to detect the distribution of CD11b+ cells in myocardial section from rats with AMI induction and injection of conditioned medium derived from OGD-treated cardiomyocytes. (**D**) Immunofluorescent staining to detect the distribution of CD11b+ cells in myocardial section from rats with AMI induction and injection of conditioned medium derived from OGD-treated cardiomyocytes with anti-miR-21 pre-transfection. (**E**) Calculation of CD11b+ cells in myocardium. SC, sham control. AMI, acute myocardial infarction. CM, conditioned medium derived from OGD-treated cardiomyocytes. Anti-miR-21-CM, conditioned medium derived from cardiomyocytes pre-transfected with anti-miR-21 (20 nM). Groups with different symbols (*, †, ‡), *p* < 0.05. n = 8 for each group. Scale bar in left lower corner indicates 50 μm.

To examine the effect of CM-OGDCM in immunomodulation after AMI, immunofluorescent staining with antibodies against CD68 (Figure 8) and CD11b (Figure 9) was performed to detect the distribution of macrophages and neutrophils, respectively. Twenty-eight days after induction of AMI, dramatically increased numbers of CD11b+ and CD68+ cells were observed in the infarcted and peri-infarcted area. Administration of CM-OGDCM reduced the number of CD11b+ and CD68+ cells in the peri-infarcted area, while the effect of CM-OGDCM in reducing immune cell infiltration was abolished by anti-miR-21 inhibition pre-transfection. 

## 4. Discussion

In the present study, we analyzed the myocardial-disease-associated miRNA expression profiles in ventricular myocardium, primary cardiomyocytes, and primary cardiac fibroblasts under ischemic stress (Figure 1). Compared with that in the normal condition, expression levels of miR-21 in cardiomyocytes and cardiac fibroblasts were up-regulated by oxygen–glucose deprivation (OGD) (Figure 1). It is of importance that the level of exosomal miR-21 in CM derived from cardiomyocytes was also up-regulated by OGD. To further determine the role of miR-21 in conditioned medium (CM), CM collected from OGD-treated cardiomyocytes with anti-miR-21 pre-transfection was applied to cardiomyocytes with oxidative stress and cardiac fibroblasts with hypoxia treatment. Results showed that inhibition of miR-21 reduced the protective effect of CM on cardiomyocytes against oxidative stress (Figure 3) and activation of cardiac fibroblasts (Figure 4). In addition, inhibition of miR-21 in CM also reduced the CM-enhanced angiogenic activity of endothelial cells (Figure 5). Rodent models with acute myocardial infarction (AMI) also indicated that administration of CM derived from OGD-treated cardiomyocyte reduced myocardial infarction (Figure 6) and collagen deposition (Figure 7) in rats with AMI induction, while pre-transfection of anti-miR-21 abolished the protective effect of CM. In addition, infiltration of macrophages and neutrophils in infarcted myocardium was also reduced by CM with miR-21 expression (Figure 8 and Figure 9).

Ischemic preconditioning is considered a physiological mechanism to protect myocardium against ischemic injury. A brief period of ischemia has been shown to protect the heart from more prolonged episodes of ischemia, reducing infarct size, attenuating the incidence and severity of reperfusion-induced arrhythmias, and preventing endothelial cell dysfunction [33,34]. In our previous study, we demonstrated that the conditioned medium derived from cardiomyocytes of ischemic myocardium contributed to reducing myocardial apoptosis and inflammation in a rat model with AMI [12]. However, the pivotal molecule for mediating the protective effects of conditioned medium has not been determined. In the past decade, secreted miRNAs have been indicated to participate in intercellular communication among different types of cells [25,35,36]. Several exosomal miRNAs, such as miR-21, miR-30a, and miR-133a have been indicated to be associated with the initiation and progress of myocardial disease [23,32,37]. In addition, recent studies have also indicated that the circulating levels of miRNAs are associated with cardiovascular disease, and can be considered a clinical biomarker for heart failure, cardiac hypertrophy, and coronary disease [29,38,39]. However, whether those miRNAs participate in ischemic preconditioning and provide benefits against myocardial infarction remains unknown. Hence, in the present study, we further examined the myocardial-disease-associated miRNA expression profiles in ventricular myocardium, neonatal cardiac fibroblasts, neonatal cardiomyocytes, and cardiomyocyte-derived CM in normal condition and under ischemic stress. Our experimental results indicated that the expression levels of miR-21 were upregulated by ischemic stress in both types of cardiac cells, as well as in the cardiomyocyte-derived CM. Previous studies have demonstrated that miRNA participates in myocardial fibrosis and anti-apoptosis of cardiomyocytes [32,40,41,42,43]. It is reasonable to suggest that miR-21 may have a role in mediating the protective effect of cardiomyocyte-derived CM against ischemic stress.

Several intracellular signaling pathways and proteins, such as PTEN/AKT/Bcl-2, TLR4/NF-κb, and PDCD4, have been found to be associated with miR-21-mediated anti-apoptosis in cardiomyocytes under ischemic or ischemia-reperfusion injury [32,40,43]. In the present study, we also demonstrated that the increased expression level of PDCD4 went along with the reduction of miR-21 in cardiomyocytes (Figure 2), while depletion of miR-21 in CM reduced its effects in protecting cardiomyocytes against oxidative stress (Figure 3). It is of note that the rat model with AMI also indicated that miR-21 in CM played a pivotal role in reducing myocardial infarction (Figure 6). In addition to preventing apoptosis in cardiomyocytes, the reconstruction of the microcirculation system is also considered a critical step to reserve cardiac function after ischemic injury [44]. Recent studies have also demonstrated that miR-21 has the ability to enhance angiogenesis, and participates in neoplastic process and neovascularization after ischemic injury [45,46]. In this study, through in vitro angiogenic activity assay, we observed that miR-21 mediated the effect of cardiomyocyte-derived CM in enhancing the angiogenesis of endothelial cells. Taken together, through the effect in protecting cardiomyocytes against oxidative stress and in enhancing angiogenesis of endothelial cells, miR-21 in CM plays a positive role in ameliorating myocardial injury post AMI. 

On the other hand, miR-21 has also been found to be associated with cardiac fibrosis and cardiac remodeling [47,48]. In this study, the in vitro cultured cell model indicated that cardiac fibroblasts incubated in CM derived from OGD-treated cardiomyocytes showed higher proliferative activity and up-regulated expression level of fibrotic proteins, while depletion of miR-21 in CM reduced fibrotic protein expression (Figure 4). However, the removal of miR-21 in CM did not further reduce the collagen deposition after AMI (Figure 7). It is possible that the depletion of miR-21 in CM not only reduced the effect of CM in activation of fibroblasts, but also down-regulated the effect of CM in preventing cardiomyocyte loss and in promoting angiogenesis. 

Elevated oxidative stress and infiltration of inflammatory immune cells are usually observed in myocardial ischemic injury and participate in myocardial inflammation and cardiac remodeling. Previous studies have indicated that the antioxidant responses in endothelial cells and apoptosis of macrophages could be regulated by expression of miR-21 [49,50]. In the present study, our data also indicated that the effect of CM in decreasing macrophage and neutrophil infiltration was reduced by inhibition of miR-21. In our previous study, CM derived from cardiomyocytes reduced the oxidative stress in myocardium with ischemic injury [12], however whether this anti-oxidation effect of CM is mediated by miR-21 remains unknown and further investigation is needed. 

Recently, researchers have demonstrated that the protective effect of conditioned medium derived from endothelial cells and mesenchymal stem cells against ischemic reperfusion stress in cardiomyocytes is contributed by secreted exosomes [51,52], while it is widely recognized that miRNAs could be transported within exosomes and participate in intercellular communication through fusion of exosomes with target cells [53]. In the present study, through the rat AMI model with CM administration, we further demonstrated that reduction of exosomal miR-21 in CM derived form OGD-treated cardiomyocytes impaired its function in ameliorating myocardial ischemic injury. Hence, it is reasonable to suggest that the protective effect of CM should be mediated by the exosome-packaged miR-21. Taken together, despite the effect on fibroblast activation, through contributions to anti-apoptosis, enhancing angiogenesis, and anti-inflammation, the expression of exosomal miR-21 is essential to the therapeutic efficacy of CM derived from cardiomyocyte in ameliorating myocardial ischemic injury.

## 5. Conclusions

Based on our findings in this study, we consider that ischemic stress increases the expression level of miR-21 in cardiac cells and CM derived from OGD-treated cardiomyocytes. Depletion of miR-21 in CM reduced its effects on protecting cardiomyocytes against oxidative stress, enhancing fibroblast activation, promoting angiogenesis, preventing immune cell infiltration, and ameliorating myocardial infarction after AMI.
